# Communication in Human-Robot Interaction

**DOI:** 10.1007/s43154-020-00026-1

**Published:** 2020-08-27

**Authors:** Andrea Bonarini

**Affiliations:** Department of Electronics, Information, and Bioengineering, Politecnico di Milano, Piazza Leonardo da Vinci, 32, 20133 Milan, Italy

**Keywords:** Human-robot interaction, Communication, Robot design

## Abstract

**Purpose of Review:**

To present the multi-faceted aspects of communication between robot and humans (HRI), putting in evidence that it is not limited to language-based interaction, but it includes all aspects that are relevant in communication among physical beings, exploiting all the available sensor channels.

**Recent Findings:**

For specific purposes, machine learning algorithms could be exploited when data sets and appropriate algorithms are available.

**Summary:**

Together with linguistic aspects, physical aspects play an important role in HRI and make the difference with respect to the more limited human-computer interaction (HCI). A review of the recent literature about the exploitation of different interaction channels is presented. The interpretation of signals and the production of appropriate communication actions require to consider psychological, sociological, and practical aspects, which may affect the performance. Communication is just one of the functionalities of an interactive robot and, as all the others, will need to be benchmarked to support the possibility for social robots to reach a real market.

## Introduction

Robots that are involved in communication are growing exponentially [1], and they will increase even faster as the communication abilities will open new applications. Social robots [2, 3•] used in public spaces (for instance, hotels [4, 5], malls [6, 7], airports [8, 9], hospitals), education [10•], assistance [11, 12], and personal care [13–15], co-bots [16, 17] used in production plants, but also smart toys [18] and autonomous cars [19, 20], all need to interact effectively with people.

Interaction may be based on communicative acts [21, 22], performed intentionally to produce some effect in the interacting agent(s), but also obtained by unintentional acts, since it is impossible not to communicate when a channel is open between two agents [23].

Human-robot interaction requires from both sides the exploitation of different sense channels [24], typically hearing, sight, and touch, respectively, presented in sections “The Hearing Channel: Sounds and Speech,”“The Sight Channel: Light and More,” and “The Touch Channel.” The signals should be not only produced and detected along these channels but need to be elaborated to produce information compatible with the decision function of both the human and robot agents, so that appropriate actions can be selected as a consequence of the communicative act. Interpretation could be either programmed, or learned, as discussed in “Learning to Interact.”

In communication, so also in HRI, it is important that all the signals involved on the different channels be coherent in order to obtain effective message exchange and to establish a good relationship between the interacting agents. From the point of view of robot expression, this could be obtained by considering all the limitations imposed by sensors, computational power, mechanical implementation, and the role to be played, to keep the robot coherently placed on the Mori’s curve [25], possibly refraining from trying to achieve impossible performances and making a robot able to play a role compatible with its physical, mechanical, and computational features.

The quality of communication between robot and humans should be evaluated to support the effectiveness of the robots to perform tasks where communication is needed, in particular for social robots. “Benchmarking: the “Real” Performance” section mentions some of the efforts that are being performed in this direction. They are part of a much larger set of activities aimed at providing some kind of certification of the robot’s abilities, needed to make them reach the real market, issue still open in most potential situations [26].

## The Hearing Channel: Sounds and Speech

Sounds can be used by robots to express emotional content by explicit production, as we have been used with R2D2 and BB8 robots of the Star Wars saga [27, 28•], or by exploiting the “natural” sounds that motors are producing [29].

The production of content of a speech can be done directly by text-to speech systems [30], which may require to define some structure of the dialogue a priori and to integrate the speech production in a framework for the interaction. Recently, this activity can be learned, typically by deep learning, to produce dialogue models [31]. A rich speech production may come from the use of dialogue management systems [32, 33•] that can produce or adapt the structure of the dialogue online by using rules, statistical models, or machine learning [34]. These systems are often computationally expensive and require computation that cannot be done on board but needs a connection to large systems, like Watson [35]. This solution may lead to lag issues, due to the time needed to generate the proper text to speech and to the unpredictable delays due to the network supporting the connection. Although these lags may produce non-fluent interaction, some tricks may be used to limit this issue, such as simulating thinking, either gesturally, or with a short text, or with generic interjections, like: “Ehm...”.

The speech production can include signals to convey emotion, typically through prosody [36, 37], which make the interaction more natural and linked to the context defined by the content and the situation.

Speech interpretation by a machine is knowing a great improvement in the last years, also due to the development of home assistants [38], which, again, need a connection to a provider that could interpret the speech, with the consequent possibility of lags. While question-answering dialogues have achieved a good, market-grade quality, several challenges to obtain fluent general dialogue are still open [39]. Most of the solutions are not using the traditional natural understanding procedures, based on signal analysis, phoneme detection, grammars, and text structure interpretation, but exploit deep learning models trained off-line and able to interpret many of the possible sentences, possibly integrated in dialogue management systems.

From prosody and analysis of text, it is possible to capture some emotional content of the human speech [40–42], which can be used to adapt the dialogue and the expression of the robot.

Short commands, useful in directive interaction, can also be successfully interpreted by systems on-chip [43], which can be hosted on-board without requiring network connections to providers. Recent developments on both deep learning algorithms and hardware technology are bringing on-chip also more powerful speech understanding systems, reaching with FPGA technology more than real-time performances [44, 45].

## The Sight Channel: Light and More

### Visual Interaction: Robot to Human

Robots may exploit the vision channel of people to produce messages through light, images, or motion.

Light can be emitted by LED or other light sources that by exploiting color, intensity, and rhythm may convey emotional states or messages with a content [46]. These can also be organized in matrices, which can depict eyes and mouth, providing an immediate expression of affective and attention signals.

Interaction through a screen, often a touch screen, allows to convey quite a lot of information, either textual or visual. This is a solution taken by many social robots to overcome possible issues affecting other channels. In many cases, it may seem not natural, being in front of a humanoid, for instance, to have to read from the screen on its chest and to push virtual buttons on it, but this is becoming common, giving to the robot the minor role of “screen bearer” and diminishing the feeling of rich interaction with an autonomous agent. In most cases, this is a way to circumvent the current limits of a full speech interaction but also an effective way to produce complex interactions, such as in the case of asking to select an element in a relatively long list of alternatives.

Images are most often reproduced on screens, but they can also be projected on the floor or on objects, e.g., to convey specific information about the intention of the robot [47]. Other surfaces can also be used for projection; an interesting example is the Furhat head [48], where facial expressions are projected from inside an opaline face giving it the possibility to show rich and highly believable facial and emotional movements at a cost and complexity relatively lower than physical and mechanical faces.

A last, interesting possibility for screens is to use them to show either an animated face or parts of it sometimes integrated in physical faces (e.g., [49]), with a much less natural effect than the mentioned Furhat, or even actual faces of people interacting with the user through the robot, in a telepresence experience. These robots are used to have a remote audiovisual contact with people that cannot be reached in a specific moment, as we have seen in the recent COVID-19 pandemics, and most of them do not even pretend to be more than a bare screen bearer.

Another way to exploit the visual channel of the human for the robot is to move its body or parts of it. This is very important also to accompany signals presented on other channels. It has been said that body language conveys most of the communication content in humans [23], and it is indeed important also for robots, to support their claims of animacy [50] and improve naturalness of the interaction. Most of the movements are pretending to mime as much as possible the analogous movements in humans [51•], with possible limitations given by the mechanical implementation of joints, usually different from the biological ones, which bring to inconsistency, mostly in movements (uncanny valley [25]). It is possible to exploit bioinspired movements also in non-bioinspired robot bodies [52–54], obtaining recognizable expressions [55], as it was studied for cartoon animation [56–58].

### Visual Interaction: Human to Robot

The robot has to understand the explicit and implicit communicative cues people produce with their body, mostly the affective expressions. Although many systems have been proposed to detect emotion from facial expressions [59, 60], they often have relatively low accuracy, leading to too quick shift in interpretation and quite demanding requirements both about face resolution and the expression of facial cues [61], often required to be unnaturally expanded to be recognized. Work has still to be done on body expressions [62] and on subtle cues, whose detection is also limited by sensor resolution, the learning models which cannot come with too high complexity, and the situations where interaction actually occurs, with subjects moving fast in front of the robot, and reaching positions out of the camera range.

Explicit, ample gestures can be easily detected by cameras, mostly to be interpreted as commands. More natural human activities can be recognized, at least under constrained situations not so common in social robot applications, since most of the models have been developed for surveillance or other purposes related to entertainment and require settings not all common for social robots, such as depth cameras, fixed in the environment, presence of a single user, models developed in controlled environments, and subjects distant from the camera. Moreover, only limited sets of actions have been considered in the data sets used to learn the available models, mostly by deep learning [63], and many actions interesting for HRI are not included in those sets. Reliable identification of common gestures, in a wild environment, from a mobile camera mounted on a social robot is still an open research issue.

A simpler, although less informative, interaction channel related to vision is based on low-cost range sensors (sonar, infrared), which provide the distance from an unidentified object, possibly a moving person. The analysis of the dynamics of these signals can establish interesting interaction in low-cost, low computational power applications, such as robotic toys used in games.

## The Touch Channel

Some robots rely on touch for at least some of the possible interactions.

Simple touch detectors, such as buttons, are integrated in many robotic toys, while resistive or capacitive sensors are common in many other robots, often used to detect affective gestures such as hugs, caresses, and punches (e.g., [52, 64]). More complex, distributed, and expensive sensors can be used to detect also punctual interactions, similarly to an artificial skin [65].

Manipulation of small robots can also be interpreted from accelerometer and gyroscope sensors, widely used to detect all sort of activity in smart phones and watches, thus enabling interaction through, usually implicit, communication acts. For instance, in [66], these data are used to interpret the manipulation of a plush robot intended to be used by autistic children, in order to objectively evaluate their activity and to have the robot react to undesired actions, such as being thrown to others. Accelerometers can also be used to interpret gestures or human activities. For instance, in [67], a human player involved in a robogame wears an accelerometer that gives implicitly to the opponent robot information about the type and the quality of activity that the person is performing, thus allowing the robot to adapt its playing style to the human player.

There are also situations, e.g., in robotic rehabilitation, in robogames, or in cobots, where the way the robot enters in contact with the human body should convey messages about safety and confidence, which require this channel to be effectively expressed.

## Learning to Interact

Machine learning plays a relevant role in supporting different aspects of the communication between robots and humans, in particular in supporting the basic interpretation of signals: image content from camera images, speech content from audio signal, manipulation characteristics from contact sensors, and accelerometers. Another important aspect concerns the generation of behaviors, including planning and execution.

We have already mentioned many possible aspects of HRI communication that exploit machine learning, and we only present in this section some general considerations.

### Interpreting the Signals

In most cases, signal interpretation is based on classification, today performed in many cases by using quite complex architectures belonging to the wide category of *deep learning*. In many cases, models are available to classify objects and people’s actions: they have been developed spending a large amount of time and effort, and they are regarded as general models, good for many purposes. Being all layered models, it is also possible to retain the first, more data intensive levels, and train models on specific situations, for instance, for the recognition of activities or objects not included in the original data set. Usually, the last layers start from higher level features, requiring less time and effort to be learned.

Key points for the application of deep learning in HRI are the need of extensive data sets covering all the aspects interesting for the specific application and the need of massive computational power to learn the models. While this second issue can be addressed by off-line learning activity and with the support of cloud computing and data centers, the first one is critical and leads in many situations to the use of tools and models that some member of the community kindly made available, with few possibilities of tuning or obtaining what would really be needed for the specific application. In other situations, the collection of proper data sets is simply not possible, so the need for a different learning approach is emerging, but still not addressed.

### Learning and Adaptation of Interactive Behaviors

Behaviors can be learned by following different approaches.

*Imitation learning* requires that a task be performed by some other agent (either human or another robot), and descriptions of both situation and taken actions are considered to build the new model. This approach is generalized by *supervised learning* where the proper behaviors for given situations are directly provided in their representational form.

Another promising approach is *reinforcement learning*, where some evaluation of the performance of the robot is used to promote correct actions and discard the worst ones.

To implement effective social robots, learning in the actual situations where they will operate is crucial. In particular, it is important to model not only the multimodal features describing the person the robot is interacting with, but the whole situation, including other possible persons, objects, surroundings, and possibly anticipating what will happen next [68]. Replicating the multimodal interactive actions could also be learned, e.g., modeling synchronous speech and gestures for a humanoid robot [69].

The complexity of interaction requires, mostly in long-term activities [70], to understand what are the general attitudes, personality [71], preferences, and possible limitations of the interlocutor(s) and try to match them [72]. The identification of these aspects, and consequent adaptation of the robot behavior to the specific situation, including the selection of the appropriate multimodal communicative actions, can be done by learning models of general attitudes, trying to classify the interlocutor from the interaction, to identify the type of the most appropriate modality, and to apply the learned model to generate the proper interaction. It has been observed both for verbal interaction (e.g., [73]) and for non-verbal interaction (e.g., in robotic games [74, 75]) that robots matching the characteristics of the interacting people can have better performance.

Given safety and performance issues in real environments, and the usually long learning time, needed to identify complex models general enough to be used in the desired range of situations, learning is often done in simulation, with possible issues about how much realistic could be the learning experience in a simulated world that cannot include real people, whose behavior is difficult to simulate. The same issues hold for *adversarial learning*, where two learning systems learn from each other, again in a simulated environment, the only one where it is possible to perform the needed, very large number of iterations. Also for behaviors, different learning approaches may be beneficial, at least for some aspects and for complex applications.

## Benchmarking: the “Real” Performance

“Robot benchmarking can be defined as an objective performance evaluation of a robot system/subsystem under controlled, reproducible conditions. [...] A benchmark includes a set of metrics together with a proper interpretation, allowing the evaluation of the performance of the system/subsystem under test according to well-specified objective criteria. In particular, a benchmark can be used to certify properties and functionalities, and therefore takes a key role in demonstrating the worth of specific solutions to prospective adopters, be they companies contemplating the realization of new products, or their clients interested in the purchase of such products” [76].

Benchmarking is becoming relevant also in HRI when an objective evaluation of the performance of the robot would be required to match the market requirements. HRI benchmarking activities are still in their infancy [77–79], but their importance will increase as certification processes will be defined for interacting robots, and the real market will require guarantees for value, performance, and safety.

## Conclusion

We have presented a concise list of issues concerning communication in HRI. It is evident that communication includes transmission of signals among the communicating agents as explicit, implicit, and involuntary interaction stimuli, in an integrated flow, that has to be considered as a whole.

We have left apart all considerations about physical aspects of the robots—such as shape, dimension, skin material, and weight—which are also important elements that affect communication, but would have required as much space, dedicated to robot design; also, this is knowing a new season, since social robots will face a market of people used at high-quality design.

Despite the great efforts required to make acceptable the communication with a complex device as a robot, considering the different aspects and the inherent limits involved in this activity, the scientific community facing these challenges is growing exponentially, striving to contribute to the definition of objects that could be really considered as companions in our activities.
